# Larval *Anopheles* Species Composition and Diversity at Different Habitats and Seasons of Gondar Zuria District, Ethiopia

**DOI:** 10.1155/2022/9767155

**Published:** 2022-08-08

**Authors:** Yelfwagash Asmare, Melaku Wale, Sualih Adem

**Affiliations:** ^1^Bahir Dar University, P.O. Box 79, Bahir Dar, Ethiopia; ^2^Makisegnt Secondary School, North Gondar, Ethiopia

## Abstract

*Anopheles* species' larval habitats are diversified and season dependent. *Anopheles* larvae can be found at different habitats and their preference may vary seasonally. Knowledge of species diversity and distribution helps plan malaria control interventions. *Anopheles* larvae were sampled using the WHO standard 350 ml dipper from breeding habitats of irrigation, pond, sewage, stream, and swamp. The collected sample larvae were identified microscopically to species using morphological keys. Among *Anopheles* species recorded, *Anopheles gambiae* complex was the most abundant followed by *Anopheles christyi, Anopheles cinereus, Anopheles demeilloni*, and *Anopheles pharoensis* in descending order. *Anopheles* species occurred more in January than in other months of the study period and less in March and April. For any particular mosquito species, larval abundance did not significantly vary between the habitats; in other words, all habitats contributed equally. In this study, we confirmed that *Anopheles* mosquito larval population varied more with respect to species than to habitats and months. Interventions could be launched targeting each habitat; during the month, numbers were high.

## 1. Introduction

Female *Anopheles* mosquitoes transmit deadly malaria disease-causing pathogens [1, 2]. To date, globally, about 500 *Anopheles* species are listed. Among the hundreds of identified *Anopheles* species, 30–40 species are responsible for malaria transmission, of which about 15 species are the major ones [3–5]. *Anopheles gambiae* complex*, An. funestus, An. nili, An. pharoensis,* and *An. moucheti* are malaria vectors in Africa [6]. *An. gambiae* complex and *An. funestus* complexes are the dominant malaria vector species widely distributed in Africa [4, 6]. *An. gambiae* complex accounts for the majority of malaria cases and deaths [7]. *An. arabiensis* is the major malaria vector widely distributed in Ethiopia [8–11]. *Anopheles* species have diversified larval habitats; in some cases, different species share the same habitats, while other species are more selective for habitats with specific characteristics [12–15]. Spatial and temporal distributions of *Anopheles* species larvae are affected by habitat types and environmental factors including temperature, humidity, and precipitation across seasons [16–19]. Studies reported associations between habitat types of different *Anopheles* species and their abundance varied across months [14, 16, 19–21]. However, in the current study area, there is no information on the species diversity and abundance of *Anopheles* mosquito larvae in relation to habitat types and seasons.

The objective of this study was to identify *Anopheles* species, determine their association to different habitats—swamp, pond, stream, sewage, and irrigation canal—as well as determine seasonal variation.

## 2. Materials and Methods

**Figure 1:**
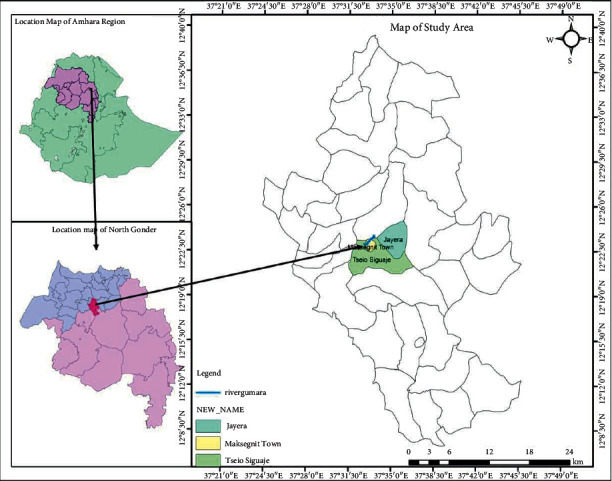
Map of the study area and sampling sites.

*Anopheles* larval sampling was made in potential breeding habitats at Gondar Zuria District (37° 24′ 24″E-37° 45′ 43″ E and 12° 7′ 23″ N-12° 39′24″N), 1500–2300 m above sea level (Figure 1). The district experiences mild temperatures from 11 to 32°C. Mean annual rainfall ranges between 900 mm and 1035 mm, which is bimodal, with short rains from March to May, long rains from June to September, post rains from October to November, and dry season from December to February [22]. Larvae of *Anopheles* species were collected using the WHO standard 350 ml dipper from breeding habitats of irrigation, pond, sewage, stream, and swamp. Sampling was conducted in the dry season (December/2019–February 2020) and the short rainy season (March–May/2020). Ten technical replicates (dips) on 10 subsites of each habitat type were made to collect *Anopheles* larvae monthly during the study period [16]. The collected larvae were preserved in 70% alcohol in separate coded vials within 24 h of collection. Species were identified using dichotomous keys and recorded with respect to habitat and season [23, 24].

### 2.1. Data Analyses

Data were subjected to analysis of variance using SAS software (version 9.4) for determining differences in larval abundance between habitats, between months, and their interaction, where appropriate. Detrended correspondence analysis was performed to determine the association between species and habitats and between species and sampling months. And finally, a number of species diversity indices were computed to establish the diversity of species at different habitats and months. Correspondence analysis and diversity indices were computed using PAST 4.03 software [25].

The following 11 diversity indices were used in the present investigation.

1. Dominance (D) = 1−Simpson's index, which ranges from 0 (all taxa are equally present) to 1 (one taxon dominates the entire community completely).
2. Simpson's index (1−D) = 1−dominance, which measures the “evenness” of the community from 0 to 1.
3. Shannon index (entropy), a diversity index, takes into account the number of individuals as well as the number of taxa. It varies from 0 for communities with only a single taxon to high values (as high as 5) for communities with many taxa, each with a few individuals. It compares the diversity between various habitats.
4. Menhinick's richness index is the ratio of the number of taxa to the square root of the sample size.
5. Margalef's richness index is used for small samples. It can be measured as *H*=(*S* − 1)/ln  *N*, where *H* stands for Margalef's index, *S* for the number of species, and *N* for the total number of individuals.
6. Equitability (also known as Pielou's evenness) is Shannon diversity divided by the logarithm of the number of taxa. This measures the evenness with which individuals are divided among the taxa present. *J* = *H*′/log(*S*), where *H* stands for Shannon index and *S* for the number of observed species in the community.
7. Fisher's alpha is a diversity index, defined implicitly by the formula *S*=*α*  ln(1+*n*/*α*), where *S* is the number of taxa, *n* is the number of individuals, and *α* is Fisher's alpha. Fisher's *α* measures diversity within a population, and it is considered the best diversity index for many communities of species, including *Lepidoptera.*
8. Buzas and Gibson's evenness: *E* *=* *e*^*H*^′*/S*, where *H* is the Shannon index, *S* is the number of species, and *E* is the measure of evenness or equitability
9. Brillouin's index is more sensitive to species abundance and is calculated as *HB* *=* ln(*N*!)—ln(*n*_*i*_!), where *HB* stands for Brillouin's index, *N* for the total number of individuals in the sample, *n*_*i*_ is the number of individuals of species *i*, and ln(*x*) refers to the natural logarithm of *x*.
10. Berger–Parker dominance: simply the number of individuals in the most dominant taxon relative to *n*. A simple mathematical expression relates species richness and abundance and takes only the commonest species in the sample. *d* = *N*_max_/*N*, where *N*_max_ stands for the number of individuals in the most abundant species and *N* is the total number of species. It expresses proportional importance of most abundant species.
11. Chao 1, bias corrected: an estimate of total species richness based on the numbers of singleton and doubleton species. Formula: chao1 = S_obs + *N*_1(*N*_1-1)/(2^*∗*^(*N*_2 + 1)), where *N*_1 and *N*_2 are the counts of singletons and doubletons, respectively.

## 3. Results

### 3.1. Distribution of Larvae at Different Habitats

During this study, a total of 10195 larvae, i.e., 2025 from irrigated, 1987 from sewage, 2030 from pond, 2008 from stream, and 2145 from swamp were collected. Similarly, a total of 3697 *An. gambiae* complex, 1992 *An. christyi*, 1638 *An. cinereus*, 1509 *An. demeilloni*, and 1359 *An. pharoensis* larvae were collected.

According to the results of the analysis of variance, while the interaction effect of habitat and larval species was significant (*F* = 33.03, DF = 24,125, *P* < 0.0001), it was not the case between months and species of *Anopheles* mosquito numbers (*F* = 0.98, DF = 16, *P*=0.4802).

None of the mosquito species significantly varied in abundance between different habitats (Table 1). Regardless of habitat, *An. gambiae* complex had the highest larval abundance, followed by *An. christyi*, *An. cinereus*, *An. demeilloni*, and *An. pharoensis*, in order of importance.

### 3.2. Seasonal Variation of *Anopheles* Larvae

According to the result of the analysis of variance, the number of larvae varied significantly with respect to months (*F* = 33.3, DF = 29,120, *P* < 0.0001). Numbers were slightly higher in January and lower in March and April (Table 2). Regardless of differences in sampling months, *An. gambiae* complex had the highest larval abundance, followed by *An. christyi*, *An. cinereus*, *An. demeilloni*, and *An. pharoensis*, in order of importance.

### 3.3. Detrended Correspondence between Mosquito Larval Species and Habitats

**Figure 2:**
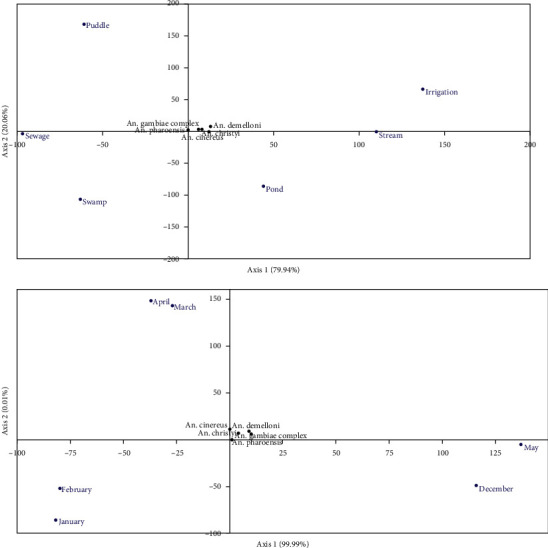
Detrended correspondence analysis between species abundance and time factor (months across the season).

In the case of habitats, the correspondence analysis explained 100% of the association between habitats and species, with 80% in dimension 1 and 20% in dimension 2 (Figure 2, top). Dimension 1 was a result of the opposite association between irrigation and sewage and dimension 2 was that of irrigation and pond or puddle and swamp. As to the species, most of them were found close to the centroid (origin) which means that they all had similar trends, although there was a tendency to align themselves on different sides of dimension 1, showing some differences between them. None of the species appeared to be associated with any of the habitats.

### 3.4. Detrended Correspondence between Mosquito Larval Species and Months

The correspondence analysis generally accounted for 100% of the association between species and the habitats (Figure 2, bottom). Dimension 1 accounted for 99.9% of the association between time (month) and a number of individuals of each *Anopheles* species corresponding to January and February (left) vs. May and December (left). Dimension 2 explained nothing for the association.

Most of the *Anopheles* species appeared to be spaced not very far from the origin, indicating a similar contribution and none of them had an affinity to any month.

### 3.5. Diversity Indices

According to the results of the Shannon–Weiner index, the species diversity of *Anopheles* mosquito larvae appeared to be similar across the different habitats (around H′ = 1.5). This happened because all habitats had the same number of species, i.e., 5. There was no dominance of any species in any habitat during this study (*D* = 0.23). A similar condition was found for Simpson_1_D across the different habitats. Equitability (*J*) was high indicating that species were equitably represented in all habitats (*J* = 0.94). Other indices did not vary across the different habitats (Table 3).

When the same thing was seen for sampling date across the season, a similar pattern was observed as the one seen on habitats, with negligible differences (Table 4).

## 4. Discussion

In this study, we confirmed that the different habitats had similar abundance of *Anopheles* mosquito larvae, while the different mosquito species varied in abundance significantly, for example, *An. gambiae* complex was the most abundant of all.

The field collection of *Anopheles* larvae in the current study indicated the presence of *Anopheles* species including *An. gambiae* complex, presumably *An. arabiensis, An. christyi, An. cinereus, An. demeilloni*, and *An. pharoensis.* Among the mosquito species recorded, *An. gambiae* complex was the dominant species and it had a stronger affinity to the stream habitat than the other habitats.

According to the detrended correspondence analysis, none of the mosquito species in the study area were found close to any of the habitats. Therefore, the present study indicated that all natural habitats contributed almost equally and no habitat was particularly associated with any of the mosquito species.

In line with the present study, various workers reported that *An. gambiae* complex was the most abundant species in different parts of Africa and Ethiopia [4, 6, 7, 26–29]. The better occurrence of the *Anopheles* species in January could be because the low temperature of the study area is not optimum for frequent adult emergence and the larvae stay a long time in breeding habitats [17–20, 30].

Similar to the present study, the larvae of *An. gambiae* complex prefers small sun-exposed habitats mostly associated with slow-moving streams due to the comparative stability of the habitats with the optimum temperature where the female mosquito prefers to lay her egg and the eggs hatch in these conditions [19, 20, 27, 31, 32].

In contrast, Hinne et al. [29] reported that ponds of dug-out wells were the most productive habitats of *Anopheles* mosquitoes during the dry season, while swamps were also productive both in dry and rainy seasons in Africa. On the other hand, Getachew et al. [27] reported no association between the primary malaria mosquito, *An. gambiae* complex with swamps. Tire tracks, puddles, and collected rainwater supported the breeding of *Anopheles* mosquitoes temporarily [27].

On the other hand, irrigation-associated habitats were the major sources of *Anopheles* mosquitoes such as *An. arabiensis* and *An. pharoensis* [33, 34]. According to previous reports, *An. christyi*, *An. cinereus*, and *An. demeilloni* were mostly found in streams and swamps, whereas *An. pharoensis* were associated with swamp habitats [32].

In the current study, *Anopheles* species diversity was found to be average, i.e., H′ = 1.5, indicating some signs of environmental degradation that failed to support more diversity. In another study carried out in Mara River and its tributaries, Kenya and Tanzania, Dida et al. [19] reported lower diversity than the current one. As a result, in the current study, no species were found particularly dominant (dominance=0.24 overall), which is also supported by the high evenness and equitability values (>0.9) indicating the lack of any dominant species.

In the current study, it was possible to underpin that it was not the habitats that mattered but the species themselves that dictated mosquito larval abundance because the larval abundance of any mosquito species did not vary between habitats. Significant differences in abundance were observed among the different species because of their intrinsic differences in their bionomic potential. Abundance slightly varied between months, whereby numbers were more in January than in March and April. Further study is required to find out the year-round species composition of *Anopheles* mosquitoes and molecular identification of sibling species of *An. gambiae* complex in the area. Moreover, further study is required to determine the insecticide resistance level of the primary malaria mosquito, *An. gambiae* complex at a broader geographic area.

## 5. Conclusion

The present study gave information about *Anopheles* species diversity at different habitats during the different months. The results indicate that permanent and semipermanent larval habitats exist for malaria transmission, where vector management tools could be applied. Seasonal variation was noticed as larval population declined in March and April. *Anopheles* species diversity was rather low (about H′ = 1.5) as well as dominance (about 0.24 across all habitats and months). As a result, evenness and equitability were high (above 0.9) indicating the lack of any dominant species. The detrended correspondence analysis showed associations between habitats and or months, but none of the mosquito species had an affinity for any habitat or month.

## Figures and Tables

**Table 1:** Mean of the number of larvae at different habitats.

Taxa	Habitats				
	Irrigation	Pond	Sewage	Stream	Swamp
*An. gambiae* complex	59.16^a^	57.16^a^	70.50^a^	56.50^a^	65.83^a^
*An. christyi*	33.83^bc^	28.83^b–e^	37.33^b^	31.83^b–d^	35.50^bc^
*An. cinereus*	27.50^b–e^	27.66^b–e^	28.16^b–e^	27.16^b–e^	29.16^b–e^
*An. demeilloni*	27.50^b–e^	23.66^b–e^	26.16^b–e^	25.33^b–e^	24.16^b–e^
*An. pharoensis*	16.83^e^	18.50^de^	24.16^b–e^	17.00^e^	22.6^c–e^

Means followed by the same letter(s) are not significantly different according to the Tukey honestly significant difference test at *α* = 0.05.

**Table 2:** Mean of the number of larvae in months of the study season.

Taxa	December	January	February	March	April	May
*An. gambiae* complex	68.4^a^	68.2^a^	62.0^ab^	53.2^bc^	52.4^bc^	66.8^ab^
*An. christyi*	36.2^de^	39.2^cd^	32.4^d–f^	29.2^d–g^	29.4^d–g^	34.4^d–f^
*An. cinereus*	26.8^d–h^	30.4^d–f^	28.8^d–h^	27.8^d–h^	27.0^d–h^	26.8^d–h^
*An. demeilloni*	27.6^d–h^	25.6^d–h^	25.4^d–h^	21.8^e–h^	22.2^e–h^	29.6^d–f^
*An. pharoensis*	21.6^e–h^	21.4^f–h^	25.8^d–h^	14.4^h^	14.8^g–h^	21.0^f–h^

Means followed by the same letter(s) are not significantly different according to the Tukey honestly significant difference test at *α* = 0.05.

**Table 3:** Diversity indices of *Anopheles* mosquito larvae in different habitats.

	Irrigation	Pond	Sewage	Stream	Swamp
Number of species found (taxa)	5	5	5	5	5
Individuals	989	935	1118	947	1064
Dominance_D	0.2371	0.2375	0.2427	0.2358	0.2399
Simpson_1−D	0.7629	0.7625	0.7573	0.7642	0.7601
Shannon_H	1.523	1.526	1.516	1.527	1.521
Evenness_e^H/S	0.9175	0.9201	0.9108	0.921	0.9156
Brillouin	1.51	1.512	1.504	1.513	1.509
Menhinick	0.159	0.1635	0.1495	0.1625	0.1533
Margalef	0.58	0.5847	0.5699	0.5837	0.5739
Equitability_J	0.9465	0.9483	0.942	0.9489	0.9452
Fisher_alpha	0.6876	0.6938	0.6744	0.6923	0.6797
Berger–Parker	0.3589	0.3668	0.3784	0.358	0.3712

**Table 4:** Diversity indices of *Anopheles* mosquito larvae across the season (months).

	March	April	May	December	February	January
Taxa_S	5	5	5	5	5	5
Individuals	732	729	893	903	872	924
Dominance_D	0.2397	0.2376	0.2408	0.2433	0.2313	0.2409
Simpson_1−D	0.7603	0.7624	0.7592	0.7567	0.7687	0.7591
Shannon_H	1.517	1.522	1.519	1.514	1.541	1.517
Evenness_e^H/S	0.9113	0.9161	0.9138	0.909	0.9339	0.9119
Brillouin	1.499	1.504	1.505	1.5	1.526	1.503
Menhinick	0.1848	0.1852	0.1673	0.1664	0.1693	0.1645
Margalef	0.6064	0.6068	0.5887	0.5877	0.5908	0.5858
Equitability_J	0.9423	0.9455	0.944	0.9407	0.9575	0.9427
Fisher_alpha	0.7223	0.7228	0.6989	0.6977	0.7016	0.6951
Berger–Parker	0.3634	0.3594	0.374	0.3787	0.3555	0.369

## Data Availability

The data used to support this study are available from the corresponding author upon request.
